# Development of adolescent mental health services in Sri Lanka

**DOI:** 10.1192/bji.2022.32

**Published:** 2023-05

**Authors:** Onali Bimalka Wickramaseckara Rajapakshe, Mohapradeep Mohan, Swaran Preet Singh

**Affiliations:** 1 MBBS, MSc, MD, Consultant Community Physician, National Programme for Tuberculosis Control and Chest Diseases, Ministry of Health, Public Health Complex, Narahenpita, Western Province, Sri Lanka. Email onalir@yahoo.com; 2 PhD, Research Fellow, Division of Mental Health and Wellbeing, Warwick Medical School, University of Warwick, Coventry, UK; 3 MBBS, MD, DM, FRCPsych, Director, Centre for Mental Health and Wellbeing Research, Warwick Medical School, University of Warwick, Coventry, UK

**Keywords:** Sri Lanka, adolescent psychological problems, adolescent mental health, mental health service development, psychological issues among youth

## Abstract

Sri Lanka has faced two major catastrophes in recent history: the civil war (1983–2009) and the tsunami (2004). Furthermore, there is a continuously changing socioeconomic situation which is becoming ever more challenging. Nearly a quarter of the Sri Lankan population is a youth or adolescent, and this age group is particularly vulnerable to adversity. Over the past decade Sri Lanka has acknowledged the need to support these young people and embarked on developing adolescent mental health services, but they require further expansion. This article provides a critical review of the state of current adolescent mental health services in our country and makes suggestions for improvement.

Over the past few decades, our country has faced two major catastrophes: the tsunami disaster in 2004 and the protracted civil war (1983–2009). It has one of the highest suicide rates in the world, in the range 30–100 per 100 000 population in males and 20–70 per 100 000 population in females.^1^ Sri Lanka has an adolescent and youth population (aged 10–24 years) of 5.2 million, representing nearly a quarter of all inhabitants (22.2 million).^2^ A substantial proportion of this age group has attempted suicide (7.0%) and many report experiencing interpersonal violence (35.0%).^3^ There is an urgent need to review Sri Lanka's provision of mental health services for adolescents.

## Development of Sri Lanka's mental healthcare system

During ancient times, Sri Lanka practised Ayurvedic medicine, but this was gradually overtaken by a Western health system under British colonial rule. The first asylum to treat psychiatric patients was opened in Borella in 1847, where treatment was largely limited to occupational therapy. The formal training of psychiatrists was initiated in the 1940s and there are currently nearly 170 board certified psychiatrists in the country.^4^ A separate Mental Health Directorate was established within the Ministry of Health in 1998. The first comprehensive Mental Health Policy (2005) was developed by the Directorate, outlining new cadre categories within mental health services. These included a new Medical Officer of Mental Health at ‘Medical Officer of Health’ level of seniority, as well as community psychiatric nurses, occupational therapists and clinical psychologists. The Directorate specified requirements at each level to ensure there would be adequate training for each service category.^5^ The National Mental Health Advisory Council was formed, comprising all relevant stakeholders, to oversee the policy implementation.

According to a Service Availability and Readiness Assessment (SARA) Survey carried out in 2017, tertiary, secondary and divisional care hospitals were identified as offering ‘adequate levels of outpatient and in-ward psychiatric services’. However, child and adolescent guidance, substance misuse management, as well as support for individuals experiencing gender-based violence and elderly mental health problems, were provided by general adult psychiatrists. The contribution to these initiatives from private sector hospitals was minimal, mainly owing to a lack of trained staff. Despite the aspirations of this policy response to the Survey findings, the proposed district-level community resource centres have not been implemented so far. The provision of mental health staff has remained at suboptimal levels; only 34% of healthcare institutions have a medical officer, and only 38% a nursing officer, trained in mental healthcare.^6^

## Adolescent mental health problems in Sri Lanka

Worldwide, nearly one in seven adolescents has a mental health problem (mainly depression, anxiety and behavioural disorders) and these contribute to a substantial proportion of the global burden of mental disorders. Among older adolescents (15–19 years) suicide is the fourth leading cause of death.^7^ Overall, population data from Sri Lankan hospitals providing mental health services has identified that depressive disorders, disorders due to psychoactive substances and schizophrenia are the foremost reasons for seeking treatment,^8^ but comprehensive data on youth mental health are not available as the country lacks surveillance data on this population.

A national school-based student health survey in 2016 reported that 38.6% of school children aged 13–17 had experienced bullying within the preceding month. Nearly 9.5% had seriously considered attempting suicide.^3^ The high risk of mental health problems was associated with factors such as the misuse of alcohol (20%) and narcotic substances (12.7%) among those 15–24 years of age. One in five reported worries about financial difficulties, educational and occupational dissatisfaction and relationship problems, and 6.4% had seriously attempted suicide.^9^ A further complicating factor is that Sri Lanka loses around 200 000 of its population annually owing to emigration; 75% of migrating females were married and most of them had children. Mental health problems are significantly higher among children from these families.^10^

During the civil war (1983–2009), the Liberation Tigers of Tamil Eelam (LTTE) and other Tamil militant groups were known to use youths in their armies. In parallel, there were large-scale recruitments into Sri Lankan government forces too, and these were also targeted at young people.

Fighting in both armies led to severe physical and psychological trauma. Youths who lived in conflict areas suffered from a lack of security, a fear of forced recruitment into the LTTE, displacement, as well as a lack of basic needs. These included food, healthcare and adequate sanitation. There was an economic breakdown, which in turn gave rise to many psychological problems.^11^ Outside the immediate conflict zones, young people in other areas of the country experienced suicide bombings, the risk of loss of their parents and loved ones, and exposure to mutilated bodies. Their sense of security was constantly challenged because of the frequent military checks and evacuation drills in schools; they grew up in an environment of uncertainty and fear.^12^

Then there was the tsunami on 26 December 2004, which resulted in 850 000 displaced people. The death toll of around 35 000 affected nearly 240 000 families across the island. In the wake of the tragedy and its impact on societal infrastructure, the population's psychological and emotional needs were poorly addressed. The subsequent impact on adolescent mental health was considerable, with problems affecting over 40%, according to some surveys, owing to the loss of family members and close friends, displacement and financial consequences, and a negative impact on school performance.^13^

## Development of adolescent mental health services

Sri Lanka's 2005 Mental Health Policy identified child mental health services as a specialty that needed to be further developed.^5^ The policy was implemented within the National Mental Health Action Plan, which had been developed by the Mental Health Directorate and experts in psychiatry. However, there was little specific reference to adolescent mental health services. In the capital Colombo and in the central city of Kandy, in-patient child psychiatry units were established in 2002 and 2014 respectively. The first adolescent in-patient unit was opened in 2016 at the National Institute of Mental Health, Colombo. Currently, few psychiatric beds in the country are allocated to children (just 12) and only 9 beds are reserved for adolescents. These services are limited to certain districts, and many of the children and youth admitted to them are managed by general adult psychiatrists. Concerns have been raised about the use of debatable practices such as pharmacological treatments designed for adults being used to treat children and adolescents.^4^ Owing to the limited number of dedicated beds for children with mental health problems, they may be admitted to other units, a deviation from the 2005 policy, which required children needing admission to be placed in local paediatric wards or children's mental health wards.^4^

## Barriers and way forward

Youth mental health services should be recognised as a separate entity from adult mental health services in Sri Lanka. It is essential to develop youth-friendly mental health services. The country's Mental Health Policy only identified children as a special category. It did not recommend the development of explicit adolescent services, although the revised mental health policy for 2020–2030 has been extended to identify the needs of adolescents. The Health Master Plan developed for 2016–2025 recommended a life course approach to mental health, focusing on specific age groups, including youth.^8^ This initiative will help to streamline this service category within the health service delivery structure. The Sri Lankan health system is predominantly run by the government, which provides free healthcare, and it is largely dependent on government funds. The allocation of national expenditure for the health sector is 5%, but little of that is dedicated to mental health services, so the provision of adolescent and youth mental health services suffers from regular resource and technical drawbacks. Sound planning and timely implementation of strategies is vital to make the most of allocated funds. Augmentation of national programmes with support from the third sector is limited within the current national context because there is no proper mechanism to integrate them into the healthcare system. The development of protocols to enable the third sector to contribute to national programmes would streamline and strengthen the delivery of key services. Although private sector healthcare organisations already provide limited services, there is huge potential for them to expand. However, there is a skills gap, and training in technical expertise is a major requirement. Policies that encourage the partnership of private and government health sectors would enable expertise within the government sector to be shared with the financial resources available within the private sector.

Finally, the establishment of a mental health surveillance system would enable age-stratified data to be gathered and should therefore support attempts to create comprehensive adolescent/youth mental health services in the country. While local mental health authorities strive to achieve the above goals, sharing knowledge with experienced nations would be beneficial in enabling us to overcome potential barriers.

## Conclusions

Sri Lanka is still in the phase of developing facilities to provide comprehensive child mental healthcare but has yet fully to address the objective of establishing dedicated adolescent and youth mental health services. The country is striving forward with the provision of accessible and community-acceptable psychiatric services for adults, but there are still financial and service structure barriers to be overcome to achieve the objectives outlined here.

## Data Availability

Data availability is not applicable to this article as no new data were created or analysed in this study.
